# Toward a Unified Model of Advanced Meditation, Human Development, Meditation Maps, and Transtradition Metaphors: Facing Impermanence, Suffering, and Death

**DOI:** 10.1007/s12671-025-02632-6

**Published:** 2025-07-29

**Authors:** Terje Sparby, Matthew D. Sacchet

**Affiliations:** 1 Steiner University College, Oslo, Norway; 2 Department of Psychology and Psychotherapy, Witten/Herdecke University, Witten, Germany; 3 Meditation Research Program, Department of Psychiatry, Massachusetts General Hospital, Harvard Medical School, Boston, MA USA; 4 Integrated Curriculum for Anthroposophic Psychology (ICURAP), Witten/Herdecke University, Witten, Germany

**Keywords:** Advanced meditation, Insight meditation, Human development, Stages of insight, Meditative endpoints

## Abstract

While there exists a large body of research on the possibility of measuring certain aspects of human development, what might be called inner development has mostly been neglected, in particular as it pertains to advanced meditation. A central aspect of this kind of development, which we call meditative development, is associated with access to bliss, peace, wisdom, and the reduction of suffering, which have been regarded as highly desirable or even ultimate aims of human life. The potential for such development is currently being scientifically studied and developmentally mapped. While the use of maps to guide meditation during practice has been criticized, the conceptualization of a transformative process involving a metaphorical death and rebirth, or the dissolution of an old identity and the emergence of a new one, is common across various wisdom traditions. In the meditative traditions, some maps, such as the one described by Mahāsī Sayādaw, describe this process in a way that is both highly detailed and grounded in experience. Here, we propose an outline of the process of metaphorical “death and rebirth” in advanced meditation, which may form a foundation for the scientific investigation of meditative development and support a deeper understanding of what it means to be human.

Studying human developmental processes is a central task of psychology. Early outlines of psychological development were made by Sigmund Freud, and the last century witnessed the rise of developmental studies with increasing systematicity. Pioneering work by Jean Piaget on cognitive development (Piaget, 2002) led to investigations into several other areas of human development. Research on moral development was initiated by Lawrence Kohlberg (Levine et al., 1985). Drawing on the work of Erik Erikson, Jane Loevinger described the process of ego-development (Loevinger, 1987). James W. Fowler proposed that faith also follows a specific developmental pattern (Fowler, 1981). Kohlberg, Power, and Simpson speculated that a further spiritual level of development may exist, characterized by progressing from despair when facing the finitude of existence to a sense of being connected to a greater whole, expressing inclusive and charitable agape-love, and accessing higher states of consciousness (Kohlberg et al., 1981). While spiritual and moral development may be connected, they should not be conflated; as pointed out by John C. Gibbs, the early stages of moral development may represent a naturalistic Piagetian sequence; the latter ones may represent a reflective extension of the earlier ones focused on existential themes (J. Gibbs, 1977). The idea that meditation and related higher/altered states of consciousness may have a positive developmental impact has found some support in neuroscience (Fell et al., 2010; Gervais et al., 2023; Orme-Johnson, 2000; Singleton et al., 2021; Travis, 2014).

While there may be important cultural differences within moral development (Ma, 1988), there is also evidence for cross-cultural validity (J. C. Gibbs et al., 2007; Q. Zhang, 2018). Certain aspects of cognitive maturation from a preoperational stage (e.g., learning to use words and symbols) to a formal operational stage (e.g., performing abstract inferences) seem to be inherent to the human being, although a specific cultural environment may be necessary for the inherent potential to unfold (Dasen, 2022).

The field of adult development, including the work of Robert Kegan, has studied how people continue to evolve in adulthood through different stages that are characterized by interpersonal relations, self-authoring, and transformative integration of different values and identities (Kegan, 1982), while also facing specific challenges like societal pressure to reach a certain developmental stage (Kegan, 1994), and overcoming resistance to such change (Kegan & Lahey, 2009). According to Kegan, a major arc of adult development is from the meaning making of an independent self to meaning making in relation to larger interconnected systems, including religious communities (Kegan, 1980). We believe that meditation, and advanced meditation in particular, which we will discuss in-depth below, offers a powerful unstudied aspect of adult development. Specifically, we argue that advanced meditation adult development is concerned with meaning making in relation to the meditative traditions and their developmental maps and metaphors of transformative psychological growth.

As nations and cultures become more interconnected and increasing efforts are made to support human development, including longevity, knowledge, and standard of living, across the globe, it is increasingly vital that we specify objective criteria for what constitutes such forms of human development. The World Health Organization (WHO) uses the human development index (HDI) that measures development based on years of education, life expectancy, and economic productivity (WHO, 2025). Such criteria ignore culture and inner life. While spiritual and religious development may be seen to be strongly tied to culture, certain meditative states and stages may be “hardwired” into the human being (Rose, 2018). Is it thus possible to develop cross-cultural maps that objectively measure the development of inherent spiritual and meditative capacities?

More explicitly, by capacities we mean volitional access to states and stages of effortless focus, profound bliss and peace (Brasington, 2015; Bucknell, 1993; Catherine, 2008; Chowdhury et al., 2023; Hagerty et al., 2013; Sparby & Sacchet, 2024; Yang et al., 2023a, 2023b), deep, transformative insight and wisdom (Braun, 2015; Grabovac, 2015; Sayādaw, 2016), the cessation of suffering, and the unfolding of a sense of meaning, connectedness, and pro-social qualities (Luberto et al., 2018; Yaden & Newberg, 2022). To support the unfolding of meditative development in individuals and across different populations (Galante et al., 2023; M. G. Wright et al., 2023), it is vital to research the maps of these developmental processes. Without this research, the deep potential for beneficial impact, not least on mental and physical health, but also on human flourishing, may remain unrealized.

## Research on Maps of Spiritual and Meditative Development

We define meditation as the deliberate engagement in one or more of a set of mental activities such as attention, observation, and imagination, the cultivation of which is performed not only for its intrinsic value but also to realize different secular and non-secular aims internally related to the practice itself (Sparby & Sacchet, 2022). Meditation is central to mindfulness practice (Oman, 2025) and is associated with mental health benefits (Karo et al., 2024; Khoury et al., 2013; Pan et al., 2024; X. Zhang et al., 2024) and other psychological benefits such as increased cognitive abilities (Eberth & Sedlmeier, 2012; Whitfield et al., 2022). Research is now expanding into the third wave of meditation research: advanced meditation (Sacchet et al., 2024; Sparby & Sacchet, 2025), which consists of states (e.g., concentration absorption states) and stages (e.g., the stages of insight) of deeper engagement with meditation practice that develop toward meditative endpoint events (e.g., extended cessations) and persistent psychological changes. Central research questions of advanced meditation, and in particular meditative development, include clarifying the domains of development (what are the markers of different states, stages, and endpoints?), how the domains are interrelated (does access to certain states impact other developmental trajectories?), and the process of developmental unfolding (linearly, non-linearly, etc.).

Within the context of clinical mental health conditions (e.g., mood and anxiety disorders), meditative processes are often characterized by internal regulation of attention and reactions to thoughts, emotions, and impulses. As challenging aspects and tendencies of the mind, for example, rumination, worry, and impatience, are reduced, certain positive qualities may emerge. These positive qualities, such as awareness, acceptance, and peace, develop gradually and slowly. However, a central feature of meditative development is that at a certain point in this process, intentional control over fundamental aspects of the meditative process may arise, including the ability to shift into concentrative absorption states, generate bliss, and make the mind extremely peaceful (Brasington, 2015; Bucknell, 1993; Catherine, 2008; Hagerty et al., 2013; Sparby & Sacchet, 2024; Yang, Chowdhury, et al., 2023; Yang et al., 2023a, 2023b). The process of meditative development may unfold over the course of years, and mastery of states, stages, and endpoints at first may appear during periods of intensive meditation retreat. Gradually, a meditator may gain increasing control over the process and may repeat parts of it voluntarily, also outside of intensive meditation retreat settings. This developmental process may be helped by meditation manuals (maps) that include instructions for how to develop mastery (Brasington, 2015; Sayādaw, 2016; Snyder & Rasmussen, 2009).

While some aspects of human development may be conceptualized in a linear manner, meditative development appears to involve a complex relationship between linear, non-linear, and cyclical processes. A typical unfolding of advanced concentrative absorption meditation (ACAM), also known as *jhāna* (Arbel, 2017; Hagerty et al., 2013; Quli, 2008), may consist of a breakthrough event where access to the state is first achieved in a retreat setting, and may initially only be possible to access and maintain within such settings (Sparby, 2019). The unfolding of ACAM typically follows a specific pattern where intense bliss is metamorphosed into intense internal clarity, happiness, and peace; as mastery grows, it may become possible to also move backwards through the process and jump between different ACAM states (Sparby & Sacchet, 2024). Even meditation that focuses on the stages of insight, where the meditator goes through a process of disentangling phenomena and personal identity, may include cycling through an overarching developmental pattern (Ingram, 2018). Endpoint states such as the cessation of consciousness may, with increasing mastery, be intentionally initiated, although this kind of mastery appears to be exceedingly rare (Chowdury et al., 2023; Laukkonen et al., 2023; Van Lutterveld et al., 2024). By “cessation of consciousness” what is meant is that the experience goes completely offline for a while (ranging from microseconds to hours and even days) even though the body remains in a meditative posture. When the mind comes online again, there is an afterglow consisting of, for example, extreme peace and a sense of rejuvenation. While the experience of cessation may be desirable in itself since it represents the absence of suffering, it is particularly the aftereffects that may be regarded as worth wanting. More research is needed to better understand these experiences.

Because advanced meditation includes the arising of intentional control over certain aspects of meditative development, such developmental processes may be studied intensively in laboratory settings. While developmental research is often limited to comparing people at different ages and stages of development, neuroscience may, when applied in advanced meditators, track the unfolding of a process that may take years or only appear in intensive retreats. While it is likely that the brains of long-term and advanced meditators may have strong differences compared to novices (Kurth et al., 2021, 2023; Luders et al., 2021), the shift that happens in advanced meditators when they, for instance, proceed from a non-meditative state to a deep absorption state may still be comparable to the development that a more novice meditator goes through when learning to access absorption states. To what extent this is the case is something that future studies may set out to discover.

The intentional control exhibited by advanced meditators may be both rare and dependent on certain kinds of practices aiming specifically at developing such control (Snyder & Rasmussen, 2009). However, such meditators are increasingly taking part in contributing to science by making their experience and skills available to scientific scrutiny. Hence, we are currently in a unique position to study advanced meditation, which has supported the meditative development in humans for centuries, and, with the spread of mindfulness and related practices, is currently being integrated into the daily lives of millions of individuals around the world.

## Maps of Transformative Death and Rebirth

Maps of spiritual development can be found in many wisdom traditions from around the world. Many of these ancient traditions speak of deeply transformative processes involving death and rebirth. The motif of an encounter with death and dissolution that leads to transformative insight, liberation, and a new life can, for example, be found in shamanism (Eliade, 1964), in myths (the Egyptian story of Osiris, the story of Odin in Norse mythology, the Aztec story of Quetzalcoatl, etc.), and the Greek mystery schools (Burkert, 1989). This motif can also be found in world religions, including in Hinduism, Buddhism, and Christianity. Some contemplative maps, such as the one drawn out by St. John of the Cross, contained detailed accounts of transformation through a dark night of the senses and of the soul (of the Cross, 2017).

The past century has included central contributions to our understanding of transformative processes, such as the cross-tradition mapping of patterns of internal change as represented in world myths and stories (Campbell, 1949), in religious experience (James, 1902) and in relation to psychedelics and breathwork (Grof, 1998). Indeed, the process of life and death has never only been a matter of biology for the human being; it has also always been a matter of internal transformation leading to rebirth. The notion that death may have a positive significance has been encoded into cultural stories that have provided motivation and inspiration to generations of people who have faced external and internal forces of dissolution. Death may represent the passing of something that is not beneficial (like habits, mindsets, and emotions such as hate and greed) and the arising of something positive and new (ways of being, deeper understanding, and emotions like awe and wonder). Similarly, in Christian theology, the wound of the Christ on the cross may not only represent death but also the place of rebirth (Atwood, 2006).

In the contemplative traditions, these patterns of development have been mapped in a proto-scientific manner, and provide a starting point for further refinement with the methods of modern science. In the following, we suggest an interpretation of meditative development that is informed by the metaphor of death and rebirth and speculate that some meditation maps may be understood as describing such a metaphorical process.

## Critique of Meditation Maps

Traditional meditation maps are typically not included in contemporary meditation and mindfulness interventions such as mindfulness-based stress reduction (MBSR). The reason may be that traditional meditation maps are religious and often include what might be considered non-clinical, or non-scientific terminology, but it may also, as we indicate below, be based on considering the use of meditation maps as being counterproductive. Meditation maps may, for example, create idealizations that may negatively impact a meditator’s ability to directly engage with experience. Such direct engagement is often seen as central to the effectiveness of meditation and mindfulness interventions. While this critique has some merit, we argue that meditation maps may still be useful in the context of advanced meditation.

MBSR is a set of influential meditation and related practices that has shown to be clinically effective for a number of physical and mental health conditions (Chi et al., 2018; Grossman et al., 2004; Khoury et al., 2015). MBSR has been at the forefront of the spread of meditation throughout society within the recent decades. However, MBSR does not include the use of the traditional maps of meditative development. Jon Kabat-Zinn, the founder and main proponent of MBSR, has given an account for the reasoning behind not recommending that MBSR instructors make use of maps (Kabat-Zinn, 2011): (1) The map is not the territory; (2) Maps hinder the embodiment of the curriculum and authentic communication between instructors and participants by not prioritizing the non-evaluative stance in relation to present moment experience that is the core of MBSR; (3) Maps may lead to idealizing a certain goal, which hinders the direct engagement with experience that is central to MBSR.

An argument against the first point is that maps should not and cannot be the territory. The function of a meditation map is to give an overview of a developmental process. Creating an overview can only be achieved by leaving out details. While a completely accurate map would contain all the details of a territory, such a map would be either too cumbersome or cluttered to be useful. Maps provide orientation when moving from one state or stage to the next and do so by providing general characteristics of what constitutes each state/stage and instructions for how to proceed from one to the other. There are numerous ways that a meditation map might be unhelpful or draw attention away from traversing the territory. A meditation map may be difficult to interpret, which may shift attention away from the meditation practice; it may prime a meditator to focus on certain conceptualizations of their experience rather than the experience itself, and a given map may be too influenced by the individual experiences of certain practitioners to be helpful to all. However, these are specific problems that are solvable when using a pragmatic approach that is mindful of such problems and also by further research on the development, refinement, and application of meditation maps.

A counterargument to Jon Kabat-Zinn’s second point is that while concern with the map may indeed draw attention away from meditation, the map can also be helpful in other ways. Presumably this is the reason why traditions have developed meditation maps. Meditation proficiency may include the capacity to discern skillful and unskillful use of meditation maps.

Against the third point, it may be argued that while idealization of meditation maps may represent a problem in part, so too does not specifying any goal. Ideals may provide inspiration. Without a goal and desired sense of outcome, activities may become directionless. Also, not indicating ways of realizing such developmental potential may lead meditators to concluding practice at certain, earlier, developmental stages. Avoiding a goal-based mindset may be important *while* meditating, but evaluating progress pre- or post-practice may not necessarily interfere with meditation. Finally, the “no-method” approach that Kabat-Zinn seems to prefer also sets up an ideal that may turn out to be no less problematic than other approaches. The most extreme formulation of the “no-method” approach would be to not do anything. When releasing all activities, it is likely that the meditator is confronted with a plethora of thoughts and emotions, making the ideal of not doing anything frustratingly difficult. Furthermore, practicing not doing anything requires checking whether any activities are taking place internally, which is itself an activity, and hence the ideal practice is non-practice, which dissolves the idea of practice completely and makes any activity into a meditation. Even the practice of mindfulness in the form of simply being with the present moment and not evaluating it sets up an ideal for practice, namely the ideal of being with the present moment and not evaluating, which again may lead to the same kinds of challenges as identified in relation to the extreme no-method approach.

While it may be argued that the idea is not to practice in a way that perfectly realizes an ideal, but rather to become aware of one’s thoughts, emotions, and other reactions while practicing acceptance and not evaluating anything that appears, that idea itself more or less explicitly provides a simple meditation map:

1. Attend to experience in a non-evaluating way. This will lead to becoming more aware of internal processes.
2. Practice acceptance in relation to thoughts and emotions that may appear.
3. This will in time lead to an overall increase of acceptance and other positive qualities (if formulated in the context of MBSR, this practice is then connected to endpoint of the reducing stress).

Having some fundamental ideal, or map, of how a process *should* unfold may hence in some capacity be unavoidable and always interfere with practice in some way. While detailed meditation maps may be unhelpful to novices, they may potentially support the unfolding of advanced meditation practice and to some individuals provide vital indicators.

MBSR was principally developed to address clinical concerns. As mindfulness practices become more widespread, it may be speculated that some people will start to progress toward advanced meditation; in fact, the altered states of consciousness that have been traditionally connected to advanced meditation may already be widespread (Wright et al., 2024). As people continue to practice through different phases of life, they will inevitably be confronted with various challenging human experiences, such as life crises, illnesses, and, if nothing else, death. Such challenging experiences may represent opportunities for deeper practice. In the Buddhist tradition, illness, aging, and death are regarded as “divine messengers” that may inspire deep practice (Bodhi, 2012, p. 227); since time is limited and the end of life is not far away, diligent practice aiming at “deathlessness” (Anālayo, 2023), another term for the cessation of suffering, should be started as soon as possible. Such meaning-making shifts in perspective make practices such as the contemplation of a decaying corpse (Ñanamoli & Bodhi, 1995, p. 148) and other ways of meditating on death understandable (Buddhaghosa, 2010, pp. 225–236). Having access to meditation maps may provide practitioners with inspiration and guidelines for advanced practice and hence help meditators realize the transformative potential inherent in impermanence and the sufferings of finitude, including death. However, advanced practitioners also note drawbacks of the kind of conceptualization that maps involve. For example, Ācariya Mahā Boowa advises meditators to avoid “any form of speculation or conjecture” and to not “allow thoughts of what you *should* be doing or what the results *might mean* to encroach upon the investigation” while practice is ongoing; doing this may get in the way of the unfolding wisdom, which itself knows “the correct path to follow and will understand clearly the truths that it uncovers” (Mahā Boowa, 2012, p. 39).

As these radical contemplative processes may be temporarily destabilizing, both meditation maps and the wisdom perspective of advanced practitioners may provide key insights and inspiration. One further potential is that having access to the perspectives of teachers and maps may also help avoid developmental pitfalls. Simply knowing that a process is likely to become difficult at certain points may provide encouragement or at least reduce confusion arising from a view that meditative development should unfold in a linear fashion. Furthermore, certain meditative stages may involve the dissolution of mental schemas, values, and habits that have previously constituted a person’s stable, core identity. The unraveling of these aspects of a person is a vital stage of the transformative death process described in many traditions, which necessarily occurs before a new, liberated, and vitalized human being can emerge. Previously, an overview of these processes has been offered by myths that inform the communities that maintain and carry them from one generation to the next. Maps of these processes provide meditators with important information and support as they navigate the difficult territories of psychological transformation.

The issue of meditation maps inevitably raises the issue of constructivism (Flood, 2007; Forman, 1990; Katz, 1978; Rose, 2018). To what extent does the use of meditation maps *shape* or even *create* experience and thereby potentially nullify the idea that the map accurately represents an objective or universal developmental trajectory? This is a deep and important discussion—one we wish to mostly sidestep here as it raises complex epistemological issues. The conundrum has in our view been formulated in a compelling way by McDowell (1996). If concepts (maps) fundamentally shape or create experience, how are they at all constrained? Maps without external constraints would be an example of what McDowell calls a conceptual “frictionless spinning in a void” (McDowell, 1996, p. 42). Conversely, if concepts (maps) are fundamentally different from experience, how do we account for experience being able to impact or constrain concepts at all? It seems that concepts (maps) and experience must be both distinct and yet the same. There are options for solving this dilemma such as introducing the Aristotelian idea of *perceiving concepts* or the view that experiences contain conceptual structures that constrain how they may accurately be conceptualized (mapped). In McDowell’s words: “we must conceive experiences as states or occurrences in which capacities that belong to spontaneity [thinking] are in play in actualizations of receptivity [perceiving]” (McDowell, 1996, p. 66). This idea may hold potential for developing a case for different maps offering a more or less accurate view of a “territory,” namely developmental structures inherent in experience.

## Advanced Concentration Absorption Meditation, Advanced Investigative Insight Meditation, and the Stages of Insight

Our work on ACAM has shown that it is possible to integrate different and in part contradictory maps of advanced meditation into comprehensive systems (Chowdhury et al., 2023; Sparby & Sacchet, 2024). ACAM is also currently being investigated with regards to understanding its neurological basis (Chowdhury, et al., 2023; Ganesan et al., 2024). There is reason to believe that ACAM is a fundamental human capacity regardless of the conceptual framework one employs to interpret the experiences connected to it, which range from extreme focus, bliss, happiness, profound peace, to pure space, consciousness, and forms of liminal, transformative, and fundamental awareness.

ACAM is also understood as preparing the mind for deep investigation of phenomena. ACAM is thought to lead to a mind that is very clear and neutral. This clarity facilitates a neutral and equanimous investigation of the structure of mental phenomena (Brasington, 2015; Snyder & Rasmussen, 2009; Sparby & Sacchet, 2024; Yang et al., 2023b), which may be hypothesized as leading to a reduction of biases in perceptual processing. Within Theravāda Buddhism, one will find one influential developmental stage model, namely *the stages of insight* (SoI), which is a map of the process of meditative development arising from the investigation of phenomena (Anālayo, 2022). We suggest that this process may be interpreted as a process of death/dissolution and transformation. The act of intentionally progressing through SoI is associated with what we refer to as advanced investigative insight meditation (AIIM).

The version of AIIM found in the work Mahāsī Sayādaw represents a particularly promising map of the process of transformative experience (Sayādaw, 2016). The map is a reformulation of a traditional Buddhist framework that lays out the progress of insight practice. Mahāsī Sayādaw’s version of the map has gained prominence within the context of what is sometimes called Buddhist modernism, a reform movement taking place around the turn of the twentieth century based on rationalism, secularism, democratization, universalism, and interaction with the Western world (Braun, 2013). The Mahāsī map is experientially based, relies on empirical observation of phenomena (Sayādaw, 2016, p. 383), and employs comprehensive taxonomies of the mind (Sayādaw, 2016, Appendix 1–3). The method integrates some degree of skepticism and the authority of personal experience (Sayādaw, 2016, p. 370).

These aspects resonate strongly with contemporary naturalistically oriented science and advances in systematic introspection such as micro-phenomenology (Petitmengin, 2006; Sparby, 2019, 2023; Sparby et al., 2020a, 2020b, 2021). However, in Mahāsī’s work as in Buddhism generally, there is also an emphasis that is mostly ignored in scientific contexts, namely that the investigation of phenomena is motivated by and proceeds toward the complete cessation of suffering for all living beings. Hence, AIIM represents an integration of close observation of phenomena and a fundamental ethical or spiritual concern.

It is important to note that Mahāsī’s account of SoI is one among many. Other examples include the one presented in the Visuddhimagga (Buddhaghosa, 2010) and Vimuttimagga (Upatissa, 1995). All of these maps would need to be conferred with to create a comprehensive and detailed account of SoI. A comprehensive account is not our concern here, but rather to show that the traditional maps may be helpful when attempting to formulate a theoretical view of the process of meditative development drawing on the metaphor of death and rebirth. Mahāsī’s account of SoI is not inherently better than other ones, but since it was formulated in the context of Buddhist modernism and employs a combination of observation of phenomena, taxonomies, skepticism, and the authority of personal experience, it may be more compatible with the language, concepts, and values of contemporary scientific views. In other words, the transposition from the Buddhist framework to the scientific one may be less extensive when it comes to Mahāsī, since he himself has already moved the Buddhist framework in such a direction. The hermeneutical work of connecting Mahāsī’s work to scientific ones may furthermore provide an important foundation of extending the interpretation to include accounts of SoI that are historically more distant, since it represents a nexus with strong links both to the past (the Abhidhamma) and to the future (cotemporary scientific approaches).

Mahāsī’s map contains 16 stages. It should be noted that there are other accounts of the SoI that do not identify 16 stages. For example, the Vimuttimagga describes 6 stages and the Abhidhammatthasaṅgaha, a central ancient manual of Abhidhamma in the Theravāda, describes 10. That different numbers of stages may be identified does not imply that the process is not the same. A path up a mountain may be divided into any number of stages and still be the same path. However, our concern here is not to argue that there is one path, but that it may be informative to consider a specific account of SoI when it comes to understanding meditative development. As we will indicate below, what one goes through during a process of meditative development may be highly individual even though common features of such processes may also be identified.

While influential, the SoI are not widely accepted in the Theravāda tradition. Certain Burmese and Burmese-influenced Western practice traditions, however, do regard the SoI as central (Anālayo, 2022, pp. 172–177). Some Buddhist traditions may not use maps of insight stages at all, and quite different maps of insight practice may be found, for instance, in the tradition of Tibetan Buddhism (Dorje, 2009). Hence, while commonalities within the insight process described by Mahāsī may be found among people practicing within that framework, these commonalities may not necessarily be found in other insight practices. However, this does not imply that the framework determines the experiences to the degree that they are in principle incomparable to experiences that arise in the context of other frameworks. Detailed comparative work involving a range of disciplines such as religious studies, anthropology, phenomenology, and neuroscience is necessary in order to gain a more complete picture of potential universal structures and particular differences. The questions to be explored include, for instance, the degree to which the changes experienced through insight meditation are temporary or not. While strong psychological changes may also be evident in the brain structures, that does not mean that they are necessarily irreversible. Changes may also be practice-dependent, meaning that they may disappear after a shorter or longer amount of time when practice is stopped. While this does not in principle undermine the significance of AIIM with regards to its psychological benefit, it may challenge traditional understandings that connect meditative development to the lasting cessation of undesirable traits. Again, these kinds of questions may be addressed by the multidisciplinary research approach indicated above.

## An Outline of the Map of Insight Stages

When it comes to practice, insight meditation, as characterized by, for instance, Mahāsī Sayādaw (2016), is primarily concerned with clearly observing phenomena (Grabovac, 2015). There are certain techniques, including what has been called noting (or labeling), that may help observe the perceptual processes with even more acuity. According to this practice, the phenomena of experience are internally noted based on the category of experience that they entail. Deep focus remains a foundation of this practice, as this prepares the mind for awareness of subtle processes and the ability to scrutinize such processing with detachment. As this meditation unfolds, the practitioner becomes aware of increasingly subtle interconnections between phenomena and the mind. Through continued analysis, the practitioner sees clearly, for example, how a series of intentions arise, how they are connected to the different sensory domains, and how positive or negative valence is connected to different sensations. Through insight meditation, three main principles of experience become increasingly apparent: (i) that all phenomena are inherently impermanent; (ii) that a self, a core personal identity, cannot be found in such phenomena; and, finally, (iii) that phenomena can never bring stable happiness and satisfaction, but rather are a source of dissatisfaction.

Clearly observing phenomena in accordance with these principles is thought to lead to transformation wherein the underlying flux and construction of experience enter into awareness (Laukkonen et al., 2023). At this stage, awareness grows into hyperawareness of every moment and there may be a perception of light and colors, images, and buzzing energies. This stage represents a peak meditative experience, where meditative skill and experience merge into an intense awareness of how phenomena arise and pass away. At the same time, the mind is now starting to be “pulled apart,” the fabrications of mind gradually dismantle, which may, in our view, be compared to a shamanic death process where the parts that constitute human identity begin to separate (Eliade, 1964). What follows may also, in our view, be interpreted as a dive into the underworld of the mind, “a dark night of the soul,” that will slowly refine and transform it, leading to “rebirth.”

But before that takes place, what might be described as difficult territory must be traversed. According to the Mahāsī interpretation of the stages of insight, parts of the territory are characterized by a series of terms with a negative but also potentially transformative connotation: dissolution, fear, danger, and disenchantment (Sayādaw, 2016). This may indeed be experienced as dreadful, with no “there” there, no solid ground, and structure. Through this process of disenchantment, which is associated with weariness toward worldly phenomena, the attachment to these phenomena weakens, motivated by a making renunciation more complete.

When traversing these difficult stages, it is important that the meditator continues to practice regardless of the harsh internal environment, which may make the meditation practice itself much more strenuous and seemingly less effective. In the background, in the most subtle domains of the mind, an uprooting of negative tendencies may start to take place. Habitual, subconscious patterns of mind cease to be repeated, and they are drained of their motivational “charge” by undercutting desire and aversion while gaining a clear view of how these patterns are not beneficial. Once this starts to consolidate, the mind will enter into a state of deep equanimity. The mind in equanimity is content, unattached, and undisturbed by phenomena, regardless of whether they are pleasant or unpleasant.

At this point, an encounter with “the void” behind phenomena may occur; consciousness may experience what is called a cessation, or *nirodha* in the liturgical language of Theravāda Buddhism called Pali, which means complete unconsciousness for typically a short period of time, though such cessations may last for hours or even days (Laukkonen et al., 2023). Through this process, we interpret the mind as “dying” while the body remains in a form of stasis. Traditionally, the mind encounters *nibbana* (Pali) (or *nirvana* (Sanskrit)), a realm sometimes characterized as absolute peace and sometimes as ineffable, but traditionally connected to the absence of conceptual designations, also referred to as *signlessness*, and *deathlessness* or the falling away of ignorance, greed, and hate, which implies being completely unaffected by mortality (Anālayo, 2023). In the Tibetan Buddhist tradition, the death process also implies achieving deathlessness in this way, but there is also posited an existence of a very subtle, fundamental awareness or clear light (Jinpa et al., 2020; Lott et al., 2021; Van Gordon et al., 2018). As the meditator emerges out of this realm, they will be able to clearly observe the deepest layers of how conscious experience is constructed. Subjectively, there will also be a strong sense of relief, rejuvenation, and liberation. Patterns of behavior that perpetuate suffering will be released. The mind is now free and reborn from out of the depths of existence.

While this map may be understood as representing a process that unfolds within a relatively quick time span during specific forms of AIIM, it may also be interpreted as providing a general overview of a powerful trajectory of meditative development. Since the process is based on observation of phenomena and identifying underlying principles, it has an affinity to scientific study. However, since the ensuing experiences also imply an existential confrontation with impermanence, dissolution of identity, and suffering, with the potential for transformation and ultimate liberation, the process directly involves an ethical, psychological, and spiritual dimension that has historically not been the domain of scientific research. Different forms of meditation and even psychedelic substances may incline the mind to emphasize different aspects of the process, and involve different terms, analogies, and imagery. Each journey through a transformative process may also have individual aspects. At various times it may be focused on, for example, resolution of emotional conflicts based on childhood experiences, attachment to specific personal identities that have arisen through life, encountering doubt connected to low self-esteem, uprooting negative habits of mind, or resolving specific addictions. However, certain parts of the SoI map seem more fundamental as they relate to universal aspects of human experience with which everyone will eventually be confronted. Through aging and the decay of the body, impermanence will make itself apparent; through death, personal identity will finally dissolve; through the non-acceptance of impermanence and death, suffering will be inevitable. For some, this process will be slow and gradual; for others it will suddenly present itself with great intensity, such as in the case of accidents, acute illness, and bodily trauma. Through meditation one may prepare for such misfortunes and death to some extent by going through an internal death process before it takes place physically.

## Conclusion

Further research into meditative development in adults may be characterized as relying on an interaction between three levels: (i) cross-tradition comparative analysis; (ii) research into particular maps supported by neuroscience and other established empirical methods; and (iii) investigation of aspects related to cultures/traditions and specific individual experience. This represents something new in relation to the spiritual traditions of the world. Previously, meditation maps largely existed side-by-side, perhaps driven by a need for practitioners to commit to one map and for each school to define their identity through exclusion. Furthermore, scientific methods for investigating human physiological development simply did not exist. Individual experience that did not fit a map would not be included; the maps were probably mostly developed based on the exemplary lives and the experience of religious leaders. This scientific approach may provide new opportunities in world spirituality by its methodical analysis not being bound by a commitment to a specific ontology, a worldview, or soteriological urgency.

There may also be deep conflicts among the maps that could potentially be resolved by deep reflection and investigation. A central question relates to whether at the end of a transformative process an ultimate dissolution or a discovery of a higher awareness will occur. Rather than resorting to describing the ultimate endpoints as “ineffable,” such states may be investigated with emerging first-person methods such as micro-phenomenology in combination with state-of-the-art neuroimaging (Sparby, 2015, 2017). We now can study the brains of practitioners who access meditative endpoints, which may help understand subjective reports (Laukkonen et al., 2023). At the heart of spiritual experience, there is a paradox, namely the paradox of annihilation and unification with fundamental awareness. One cannot experience unification without annihilation, but annihilation appears to make the experience of unification with signless awareness impossible (Goldstein, 2021). Rather than taking this as pointing to something ineffable, these two may be regarded as complementary; one may hypothesize that endpoint experiences may be tuned more toward either unification or annihilation, seeing both as lying on a scale where complete unification is the same as annihilation and complete annihilation the same as unification (Yaden & Newberg, 2022).

The spiritual meditative maps of the process of death and rebirth may provide a source of deep meaning. Even in the depths of loss and meaninglessness, new meaning may be discovered. This idea of death and rebirth may be the ultimate map of meaning, precisely because it incorporates the loss of meaning (the dissolution of previous identities, habits, and limited values). As such, it is also a deep source of resilience and mental health. This may even be understood as the *peak* of human mental health. The maps of transformative processes in wisdom traditions of the world may have had this as their deeper purpose: the recognition that facing and accepting death is the ultimate source of inner rejuvenation, strength, and hope in a world fundamentally characterized by all things coming apart. As Cicero stated regarding Greek mystery cults—what may have been a ritualized death and rebirth process—“we have recognized the initiatory rites [*initia* in Latin, also meaning “first principles], as they are called, as in truth the beginnings of life, and we have accepted the plan not only for living with joy but also for dying with better hope.” (Cicero, 2014, p. 168).

A direct, aware, and patient encounter with the difficult aspects of human existence may be one of the most profound sources of mental health available to humans. This may account for some of the success of MBSR and related clinically oriented meditation programs, and yet there is likely more developmental potential inherent in the spiritual maps of transformative processes. This is the domain of advanced meditation research. Meditative development may in time become a natural part of the assessment of overall human development, supported by the emerging science of advanced meditation.

## Data Availability

N/A. The manuscript is not based on data.

## Declarations

### Use of Artificial Intelligence

No AI tools were used when writing this article.
